# Collecting a set of psoriasis family material through a patient organisation; clinical characterisation and presence of additional disorders

**DOI:** 10.1186/1471-5945-5-10

**Published:** 2005-10-14

**Authors:** Annica Inerot, Charlotta Enerbäck, Fredrik Enlund, Tommy Martinsson, Lena Samuelsson, Jan Wahlström, Gunnar Swanbeck

**Affiliations:** 1 Department of Dermatology, Sahlgrenska University Hospital/Sahlgrenska, SE-413 45 Göteborg, Sweden; 2 Department of Clinical Genetics, Sahlgrenska University Hospital/Ostra, SE-416 85 Göteborg, Sweden

## Abstract

**Background:**

The aim of the present study was to describe the clinical characteristics of a population of psoriatics sampled from a patient organisation and not from hospitals or out-patient clinics. Furthermore, we wanted to compare siblings with and without psoriasis regarding the occurrence of other diseases.

**Methods:**

At the end of 1991, we initiated a project which aimed to study genetic factors leading to psoriasis. Firstly, we sent questionnaires to all the members of the Swedish Psoriasis Association. We then examined 1,217 individuals (570 with psoriasis) from 310 families, in their homes in the southern part of Sweden. All the available family members were examined clinically and asked about the course of the skin disease and the occurrence of other diseases. The eight hundred members of the proband generation were divided into two groups, with or without psoriasis, and their clinical features were compared.

**Results:**

Most individuals in this study population had a mild form of psoriasis. The siblings with psoriasis had joint complaints significantly more frequently than their siblings without the skin disease and those with joint complaints had more widespread skin disease. Among the other studied concomitant diseases (iritis, heart or hypertension disease, endocrine disease, inflammatory bowel disease and neurological disease), we were not able to find any difference. Seventy-seven of 570 persons were found to be in remission (13.5%). Females had a mean onset 2.5 years earlier than males. We were not able to find any correlation between the extent of the skin disease and age at onset. Twice as many persons with joint complaints were found among those with psoriasis than among those without, 28% versus 13%. Almost half (48%) the psoriatics who also had joint complaints had psoriasis lesions on their nails. Endocrine disorders were found in 9% of those without any allele for Cw6, but only in 1% of those who had Cw6. In fact, none of 183 Cw6 carriers had diabetes, as compared to the population prevalence of 3–5% in Sweden.

**Conclusion:**

With the exception of joint complaints, persons with psoriasis, collected from a patient organisation, did not have an increased frequency of (studied) co-existing diseases.

## Background

Psoriasis is a common, genetically determined inflammatory, proliferative disease of the skin, in which the immune system is involved in the pathogenesis. Among individuals with psoriasis, a substantial number also have some involvement of the joint system, but for other concomitant diseases the evidence of a connecting relationship to psoriasis is not clear.

Examinations of persons with psoriasis in census studies have been made by Lomholt and Hellgren [1,2]. Many studies with examination descriptions come from hospitals or out-patient clinics [2-6]. The recent study by Gudjonsson et al [7] describing clinical characteristics in 369 patients with familial psoriasis was also community based. Questionnaire studies of psoriasis were introduced by Farber et al. [8] and have been followed by many others from different countries [9-12].

At the end of 1991, we initiated a project designed to elucidate the genetic factors involved in the pathogenesis of psoriasis [13]. We sent questionnaires to all the members of the Swedish Psoriasis Association, of which it is estimated that approximately 10% of all Swedish psoriatics are members. Individuals from the families were examined and blood samples were collected. Based on this patient sample, we have previously reported on the identification of a novel psoriasis susceptibility locus on 3q21 (PSORS5; MIM 604316) through a genome wide scan [14]. Furthermore, we have created a locus-specific SNP map and we have utilised it to narrow down the size of the candidate region to < 250 kb [15]. A candidate gene, SLC12A8, is currently under investigation. The aim of the present study was to describe the clinical characteristics of this population of psoriatics. In contrast to other studies, they were sampled from a patient organisation and not from hospitals or out-patient clinics and are therefore more likely to reflect the true clinical distribution of the disease. There have been reports suggesting a relationship between psoriasis and an increased risk of cardiovascular disease [16], as well as several lifestyle factors, such as smoking, obesity and diabetes [5,17,18]. We therefore wanted to compare siblings with and without psoriasis regarding the occurrence of other diseases.

## Methods

The members of the Swedish Psoriasis Association constitute the study base. Approval was obtained from the local Ethics Committee. One of us (AI) examined 1,217 individuals from 310 different families. An analysis of the inheritance pattern from families of members of the Swedish Psoriasis Association was the reason for selecting families in which the inheritance could fit an autosomal recessive trait. We therefore selected families in which the parents did not have the skin disease (which is 64% of the material) and in which there were (in addition to the proband) one or more siblings with psoriasis. Persons living outside the Göteborg area were visited in their homes, or, if they preferred, we met them at a doctor's office in their neighbourhood. Blood samples were drawn at the same time. The majority of the examinations took place in September to May 1991–1998, in the southern part of Sweden up to a level about 150 km north of Stockholm. In families with two or more siblings, the examination rate for all family members was 93%. Some parents were not alive and a few individuals did not wish to take part in the study. Another reason for incomplete families was members living in another part of the country. The size of the families that were examined is shown in Table 1.

**Table 1:** Examined persons in all 310 families

**Size of sibship**	Families *	**Proband generation**		Parent generation	
	**N**	**N with pso**	**N total**	N with pso	N total

**1**	78	73	78	6	36
**2**	99	154	198	11	154
**3**	70	140	210	8	127
**4**	32	77	128	3	54
**5**	17	49	85	0	28
**6**	6	19	36	1	9
**7**	4	13	28	2	7
**8**	1	3	8	0	2
**9**	1	4	9	0	0
**10**	2	7	20	0	0
**Total**	**310**	**539**	**800**	31	417

*All families are not complete. This is most obvious in the small families, where only half of the individuals were examined. These families were not ideal for our genetic study, but still represent the study population in this clinic examination study.

All individuals were inspected on the scalp, the face, the elbow area, the umbilical and perianal areas, the knees, hands and feet and also in other areas where they were aware of having any skin changes. The distribution of psoriasis was recorded if the person had any active psoriasis at examination on the scalp, face, trunk, anogenital area, arms, legs, hands, feet or nails, i.e. in nine different locations. The subjects were also asked about other medical problems. In addition to spontaneous information, they were asked especially about joint complaints (both peripheral joints and back involvement), iritis, problems with heart disease or hypertension, endocrine disease (mainly diabetes or thyroid disorder), inflammatory bowel disease or neurological disease. We felt it was not possible to obtain reliable information about psychiatric or malignant disorders when meeting several members of the family at the same time in a home environment for a relatively short time. Because of the age difference between the two generations, we have chosen to compare these diseases among the groups with and those without psoriasis in the proband generation only.

In 312 of the 800 individuals in the proband generation, we had information about the presence or absence of the allele Cw6 at the HLA-C locus. In previous studies from our group [19,20], psoriatics have been analysed for the presence of the Cw6 allele using a PCR-based typing method, as previously described.

### Statistical analysis

The data were analysed using the chi-squared test with Yate's correction (comparison of two different groups). A *p *value of < 0.05 was considered significant. Some other statistical method was used when appropriate; this is mentioned in the text when applicable.

### Clinical diagnosis

For the majority of the probands, a dermatologist had already established the diagnosis. When the patient was free of skin changes at the examination, the diagnosis was based on the medical history, including the name of the dermatologist who made the diagnosis. The diagnostic criteria were the same as those used in clinical practice. Sharply demarcated, infiltrated, red skin lesions with silvery white scaling were considered to be psoriasis. Isolated nail lesions were not considered sufficient for the diagnosis. If unclear, the diagnosis was not set. Pustular psoriasis was not included, if the person did not also have psoriasis vulgaris.

## Results

### Correctness of diagnosis

During the first year of examination, three probands turned out not to have psoriasis, although they thought they had it and had joined the patient organisation. They and their family members have been excluded from the study. Apart from these three, all the probands had been given a correct diagnosis. We found 51 persons with psoriasis, where the proband had originally said that his or her relatives did not have psoriasis; twenty-five were siblings, 11 mothers and 15 fathers. The sex of one-third of the probands giving these reports was male (17/51), which is the same proportion as in the whole material (111/310). Among the relatives, there were three persons whose siblings were thought possibly to have psoriasis by the probands, but this could not be verified at the examination. Three other siblings were reported by the probands to have psoriasis vulgaris, but it turned out to be psoriasis pustulosa. All the other relatives reported by the probands as having psoriasis could be verified as having the disease.

### Age of onset and duration of psoriasis

In the proband generation, the ages at examination ranged from 16 to 79 years, with a mean of 40.5 years. The mean age for the 539 individuals with psoriasis was 40.2 years and the corresponding figure for the 261 persons without psoriasis was 41.3 years. There was no significant difference in age between men and women. Age of onset varied from the first year of life to 51 years of age. Females had a mean onset 2.5 years earlier than males (18.0 versus 20.5). The gender difference in age at onset was increased to 4 years when analyzing only HLA-Cw6-positive individuals and no difference was found for the HLA-Cw6-negative. The duration of psoriasis varied from not being aware of the diagnosis up to the age of 67 years (a woman aged 74, already examined in 1991), the mean duration being 21.0 years.

### Remission

In some instances, the person who was being studied did not have enough changes in the skin at the time of examination for the dermatologist to be able to make the diagnosis at that time-point, even though information from previous examinations (mostly by dermatologists) or pictures from earlier outbreaks indicated psoriasis. In these cases, the person was diagnosed as having psoriasis, but that it was currently in remission. Seventy-seven of 570 persons were found to be in remission, (13.5%; 95% CI: 13.5+/-2.8%), 43 women and 34 men, average age 40.2.

### Guttate psoriasis

Only 15 individuals (including two sibling pairs) spontaneously described a history of guttate psoriasis. One woman had had three guttate outbreaks. The highest age at the time of the outbreak was 40 years.

### Twins

Eleven pairs of twins were examined, four monozygotic and seven dizygotic. Two of the monozygotic pairs were discordant with respect to psoriasis (ages: 51 and 57 years) and three of the dizygotic pairs were discordant in the same respect (ages: 29, 43 and 49 years).

### Concomitant diseases

#### Joint complaints

If a person had or had had significant pain or aching localised to one or several joints (including back pain), he/she was classified as having "joint complaints", without trying to make a specific diagnosis. In a few persons who denied experiencing symptoms from joints, joint deformation like that caused by joint inflammation was seen at examination and these persons were also classified as having "joint complaints". There were more than twice as many persons with joint complaints among those with psoriasis than among those without, 28% versus 13%, and the difference was significant (*p *< 0.001). Among female psoriatics, 33% had joint complaints, while 21% of the men had joint complaints (*p *< 0.01). Nail changes were more common among psoriatics with joint complaints.

#### Iritis

In this case, there were very small numbers, with no difference between the groups.

#### Heart or hypertension disease

Considering that the mean age of these groups is around 40, low values would be expected and this is also what we found. There was no significant difference between the groups.

#### Endocrine disease

Four per cent reported a history of endocrine disorders, with no difference between those with and without psoriasis. Of the 32 persons with endocrine disorders, 19 had diabetes mellitus, 13 thyroid dysfunction (one of them had both disorders) and one had a disease of the parathyroid glands. The prevalence of diabetes mellitus among those people with psoriasis was 2%, while among siblings without psoriasis the corresponding figure was 4%. This is not a statistically-significant difference.

#### Inflammatory bowel disease

Of all the people in the proband generation, 19 individuals (3.5%) claimed to have inflammatory bowel disease. No difference was found between the two groups in the proband generation.

#### Neurological disease

Around 3% reported a history of some neurological disease. There was no significant difference between the groups in the proband generation.

Information about diseases above are summarised in Table 2.

**Table 2:** Concomitant diseases at examinations in the proband generation

	Siblings with psoriasis = 539				Siblings without psoriasis = 261			
N = 800	"Yes"	%	"No"	MV	"Yes"	%	"No"	MV

Joint complaints	149	27.7	389	1	35	13.4	226	0
Iritis	8	1.5	530	1	3	1.1	258	0
Heart or hypertension disease	36	6.7	499	4	21	10.4	181	59
Endocrine disease	20	3.7	516	3	12	5.9	190	59
Inflammatory bowel disease	13	2.4	523	3	6	3.0	196	59
Neurological disease	20	3.7	516	3	4	2.0	198	59

Numbers under "Yes" and "No" refer to individuals. For details see text. MV = missing value.

#### Alopecia areata and vitiligo

Alopecia areata at examination or in the medical history was found in six persons, three of whom had psoriasis. Vitiligo was found in nine persons; only one of them had psoriasis.

#### Location and extent of psoriasis, gender differences

The overall impression was that most people had a mild form of the skin disease. The examined probands (n = 302) had an average number of 4.4 different locations for their skin disease (men 5.1, women 3.9). Comparing these means produced a significant difference (*p *< 0.001) (Table 3).

**Table 3:** Severity of psoriasis, measured as number of locations affected at examination

	Males		Females	
	
	N	sum of locations	N	sum of locations
		average/median		average/median

302 members/probands	110	5.1/5	192	3.9/4
237 siblings/not members	135	2.8/3	102	2.3/2
All 539 with psoriasis	245	3.9/4	294	3.4/3

Male members of the psoriasis association have a more extended skin disease than females. Non members with psoriasis have fewer locations affected.

Men and women in the proband generation who were not themselves members of the Psoriasis Association had a lower average number of psoriasis locations, 2.8 and 2.3 respectively (NS). Persons with joint complaints had more widespread skin disease than those without joint complaints. This difference was highly significant (*p *< 0.001), comparing medians (5.0 and 3.0) (rank sum test).

We were not able to find any correlation between the extent of the skin disease and age, even if we divided the material into men and women.

**Figure 1:**
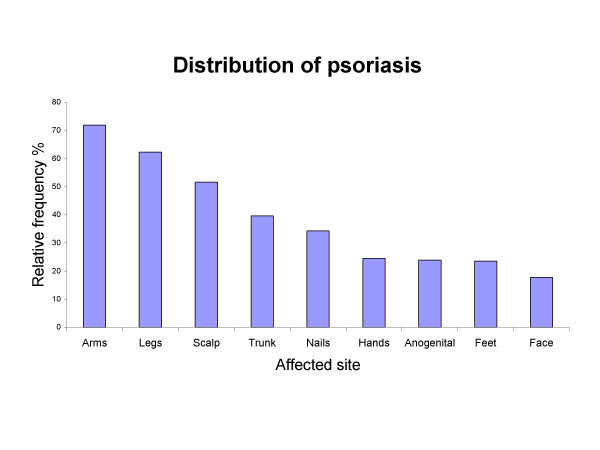
**Body distribution of psoriasis**. Body distribution of psoriasis among the 570 individuals with the diagnosis. In addition to the 539 persons in the proband generation, 31 in the parent generation, who also had psoriasis, have been included.

The most common finding was psoriasis on the arms and legs. Less common locations were the feet and the face. The anogenital area was affected in 24.3% of all the examined persons (Figure 1).

Men had psoriasis of the face more frequently than women. This difference was significant (*p *< 0.01). Men also had psoriasis more frequently on the scalp and in the anogenital area. The p-value for this difference was 0.03 for both scalp and anogenital location. The rest of the locations were distributed with no gender difference. To look for heredity in psoriasis when it came to severity, we compared 132 sibling pairs. We were not able to find any correlation between siblings from the same family and the severity of the skin disease, measured as the number of locations with involvement. Nor were we able to find any relationship between the extent of the disease and the presence or absence of an allele for Cw6.

#### Location of psoriasis and the occurrence of concomitant disease

Almost half (48%) of those psoriatics who also had joint complaints had psoriasis lesions on their nails, compared with 31% in the group without joint complaints. The chi-squared test showed that this was a highly significant difference (*p *< 0.001). A significant difference (p < 0.001) was also seen if there was anogenital psoriasis. A statistically-significant difference was also found if there was psoriasis on the trunk or the legs (*p *= 0.02). For the rest of the locations, we were not able to find any significant differences, after examining all the recorded locations and concomitant diseases.

#### HLA-Cw6

For 312 of all 800 in the proband generation, we had information about the presence or absence of the Cw6 allele at the HLA-C locus in the HLA region on chromosome 6 p. When examining other concomitant diseases, we found a difference in the prevalence of endocrine disease. This group of disorders was found in 9% of those without any allele for Cw6, but only in 1% of those who had Cw6. This difference is highly significant (*p *= 0.002), but it is based on very small numbers (11 of 119 without Cw6 and two of 183 with Cw6). Both persons with Cw6 and endocrine disorders had thyroid disease and neither had diabetes mellitus. Among the 436 for whom we had no information about the Cw6 status, but information about endocrine disease, the prevalence was 4%.

## Discussion

The psoriasis family material examined in this study was recruited from a well-informed patient organisation, i.e. the Swedish Psoriasis Association, and not, as in most other studies, from hospitals or out-patient clinics. These individuals are more likely to be representative of most persons with psoriasis than patients from hospitals. Furthermore, this family study of psoriasis is the largest in which all the individuals have been examined by one dermatologist and no previous study has compared the occurrence of concomitant diseases in siblings without psoriasis. The distribution of psoriasis was about the same as that reported by Lomholt [1,3] and Fleischer et al. [12] and partly also like that reported by Hellgren [2] and Farber and Nall [21]. The majority of the individuals had a mild disease and an estimation of the PASI score for the average psoriasis person in this study would probably not exceed 4 (theoretical range 0–72). We have used a simple method of recording any active psoriasis lesion in nine different body regions, instead of using the PASI score [22]. When it came to the extent of the skin disease, we found a difference between men and women who were members of the Swedish Psoriasis Association. However, among the siblings, we were not able to find a significant difference between men and women. As the siblings are seldom members of the association, it is probable that they are a better reflection of the country's population. Lomholt [1] found no difference between men and women when visiting people in their homes, but Molin [3], who investigated 300 former in-patients with psoriasis, found that men had a more severe skin disease, when it came to both the extent of lesions and the course. Fleischer et al. [12], who used a self-administered psoriasis area severity index score, found a significant difference between men and women, with men having a higher score. In a questionnaire survey of the Psoriasis Society of the Greater Helsinki area, Könönen et al. [9] also found more severe and more frequent skin lesions in men. In a study from Island based on 369 psoriatics, Cw6-positive patients was found to have more extensive plaques and overall disease severity [7]. However, we were not able to find any correlation between the prevalence of Cw6 and the extent of the skin disease, in agreement with previous observations on 64 patients by Ikähemo et al. [23]. A possible reason why we did not find any difference in (disease) severity correlated to the age at onset or Cw6 may be because the individuals with psoriasis were homogeneous in that most of them had very mild disease.

The overall gender difference in age at onset was 2.5 years in accordance with many other studies [3,21]. However, this difference was increased in the Cw6-positive group (4 years) and was not found at all in the Cw6-negative group in accordance with the observations of Gudjonsson et al [7].

There are not many reports of periods of remission in psoriasis. We found that 13.5% (77/570) were free of disease at examination. Lomholt [1] found 11% (28/252), Nevitt and Hutchinson [24] 9% of 75 examined and, in a Danish study of twins [25], 13 of 48 (26%) were free from skin lesions at examination.

We felt it was not possible to obtain reliable information about psychiatric or malignant disorders when meeting several members of the family at the same time in a home environment for a relatively short time. Some individuals reported atopic dermatitis in the group both with and without psoriasis, but we did not ask specific questions about this diagnosis and we found this information unreliable for analysis.

Eleven pairs of twins among 800 siblings are in the range of what can be expected. About half of them were concordant with respect to psoriasis, among both monozygotic and dizygotic pairs. This is too small a number to draw any conclusions about the heredity of psoriasis.

It is difficult to compare the frequency of co-existing or concomitant diseases with that in other studies, as the study design varies a great deal. Probably the most important factor that can result in very different figures is the selection of the study population. The study by Lindegård [26] reflected the population of psoriatics requiring hospitalisation in the 1970s and this cannot be compared with the main population of psoriatics at the present time. In a small sample of 33 consecutive psoriatics in our own investigation, only two had been hospitalised because of their skin disease. It is our impression that this is also reflected in the whole of our material.

The problem of defining a concomitant disease adds to the selection problem when attempting to compare different studies. This is illustrated by the many different prevalence figures relating to joint complaints and/or arthritis in connection with psoriasis [27,28]. We found that 28% of psoriatics and 13% of their siblings without the skin disease had joint complaints, including back pain. In our study, women had significantly more joint complaints than men, which has previously been shown by Farber et al. [8]. We cannot rule out the possibility that there might be individuals with rheumatoid arthritis in our "joint complaints" group, especially among women. The fact that joint complaints are more common if the person has psoriatic nail lesions has been known for many decades [29]. Previous investigations have also shown a relationship between the occurrence of joint complaints and the presence of psoriasis on the buttocks and in the groin [11] and between a genital localisation of psoriasis in men and arthritis [3]. Our observation that there are more joint complaints if there are skin lesions in the anogenital area lends further strength to these observations. We also found that there was an association between the extent of psoriasis (number of locations) and joint complaints, as previously observed by several investigators [3,9,12,30]. Lomholt [1] found only four persons with psoriasis arthritis among 253 native Faroese with psoriasis. He found no one with diabetes mellitus and stated that, in his clinical practice, he also very seldom found diabetes together with psoriasis. The average age of his studied population was around 10 years less than that of the proband generation in our investigation. We now know that the presence of Cw6 is associated with the early onset of psoriasis [19,20]. and we can therefore speculate that many of the psoriatics in Lomholt's study could have had an allele for Cw6. In our material, none of the persons with a known presence of Cw6 had diabetes mellitus. If Cw6 protects from diabetes mellitus, this could perhaps be a reason why psoriasis vulgaris has stayed in the population. However, it is of importance to add that Cw6 might not be the primary association in the HLA region. It has not been made clear whether the association reflects the fact that Cw6 is the true disease-causing allele or is due to linkage disequilibrium with a nearby located gene. Like Farber et al. in 1968 [8], we found a 2% prevalence of diabetes mellitus in the proband generation with psoriasis. In a later report from the same author [8] comprising more than 5,600 patients, a total of 3.5% of the psoriasis patients reported having diabetes mellitus. The prevalence of diabetes mellitus in the general population in Sweden is reported to range from 3–5% [31,32]. In our study, thyroid disorders were found in 13 individuals (= 1.6%). By way of comparison, a study in Malmö [33] found thyroid disorders in 7.9% of a population aged around 45–50 years (men 1.5%, women 14.1%).

In the large hospital-based data register from Kiel, Henseler & Christophers found more diabetes, heart failure and obesity in psoriatics, compared with age-matched patients with other skin diseases [5]. We were not able to verify this in our study population. Our findings are in agreement with what was found in the case of psoriasis out-patients in a large register study from Sweden [16]. When it came to hypertension and heart disease, we found a prevalence of 6–10% in the proband generation (mean age: 40). In the case of unspecified "heart disease", it is difficult to find figures for comparison in previous studies among the general population, but, in a study of about 7,000 middle-aged men, Eriksson & Lindgärde [33] found that 5% were receiving drug treatment for hypertension. Psoriasis in-patients both in Finland (1973–1984) [17] and in Sweden (1964–1995) [16] had an increased mortality rate compared with the general population. In Finland, analyses of the causes of death revealed excess mortality related to alcohol and smoking. In the parent generation, only five persons (1.2%) reported having inflammatory bowel disease, compared with 3.5% in the proband generation. It is known [34] that this group of diseases has increased in the population and the age at onset is often below 40 years, but the prevalence in the general population is reported to be below 1% [34-36].

In conclusion, we have analysed a large set of family material comprising psoriasis patients, healthy siblings and their parents collected through a patient organisation. The clinical features and the presence of other diseases were recorded and compared. All the individuals in the study were examined by the same dermatologist. It is likely that this study design provides a better representation of psoriasis in general than when patients collected from hospital cohorts are used. The patient material characterised in this study has been used for the identification of the PSORS5 gene locus and it will be the subject of further genetic and clinical study in the near future.

## Competing interests

The author(s) declare that they have no competing interests.

## Authors' contributions

GS initiated the psoriasis genetic project and together with JW and TM designed the study. CE performed the genetic analysis. AI examined the persons in the families and drafted the manuscript. All authors contributed and approved the final manuscript

## Pre-publication history

The pre-publication history for this paper can be accessed here:


